# A recombinant herpesviral vector containing a near-full-length SIVmac239 genome produces SIV particles and elicits immune responses to all nine SIV gene products

**DOI:** 10.1371/journal.ppat.1007143

**Published:** 2018-06-18

**Authors:** Young C. Shin, Georg F. Bischof, William A. Lauer, Lucas Gonzalez-Nieto, Eva G. Rakasz, Gregory M. Hendricks, David I. Watkins, Mauricio A. Martins, Ronald C. Desrosiers

**Affiliations:** 1 Department of Pathology, Miller School of Medicine, University of Miami, Miami, Florida, United States of America; 2 Institute of Clinical and Molecular Virology, Friedrich-Alexander-Universität Erlangen-Nürnberg, Erlangen, Germany; 3 Wisconsin National Primate Research Center, University of Wisconsin-Madison, Madison, Wisconsin, United States of America; 4 Department of Cell and Developmental Biology, University of Massachusetts Medical School, Worcester, Massachusetts, United States of America; Vaccine Research Center, UNITED STATES

## Abstract

The properties of the human immunodeficiency virus (HIV) pose serious difficulties for the development of an effective prophylactic vaccine. Here we describe the construction and characterization of recombinant (r), replication-competent forms of rhesus monkey rhadinovirus (RRV), a gamma-2 herpesvirus, containing a near-full-length (nfl) genome of the simian immunodeficiency virus (SIV). A 306-nucleotide deletion in the *pol* gene rendered this nfl genome replication-incompetent as a consequence of deletion of the active site of the essential reverse transcriptase enzyme. Three variations were constructed to drive expression of the SIV proteins: one with SIV’s own promoter region, one with a cytomegalovirus (cmv) immediate-early promoter/enhancer region, and one with an RRV dual promoter (p26 plus PAN). Following infection of rhesus fibroblasts in culture with these rRRV vectors, synthesis of the early protein Nef and the late structural proteins Gag and Env could be demonstrated. Expression levels of the SIV proteins were highest with the rRRV-SIVcmv-nfl construct. Electron microscopic examination of rhesus fibroblasts infected with rRRV-SIVcmv-nfl revealed numerous budding and mature SIV particles and these infected cells released impressive levels of p27 Gag protein (>150 ng/ml) into the cell-free supernatant. The released SIV particles were shown to be incompetent for replication. Monkeys inoculated with rRRV-SIVcmv-nfl became persistently infected, made readily-detectable antibodies against SIV, and developed T-cell responses against all nine SIV gene products. Thus, rRRV expressing a near-full-length SIV genome mimics live-attenuated strains of SIV in several important respects: the infection is persistent; >95% of the SIV proteome is naturally expressed; SIV particles are formed; and CD8+ T-cell responses are maintained indefinitely in an effector-differentiated state. Although the magnitude of anti-SIV immune responses in monkeys infected with rRRV-SIVcmv-nfl falls short of what is seen with live-attenuated SIV infection, further experimentation seems warranted.

## Author summary

Given the magnitude and impact of the HIV/AIDS pandemic, development of a safe, effective vaccine against HIV remains a top priority for biomedical research. While live-attenuated strains of the simian immunodeficiency virus (SIV) have shown promise in monkey studies, concern for safety has limited efforts along these lines. In an attempt to mimic the epitope presentation, epitope coverage, and persistence of live attenuated SIV, we have generated recombinant strains of rhesus monkey rhadinovirus (RRV; a gamma-2 herpesvirus) containing a near-full-length genome of SIV. The near-full-length genome retains 96.7% of the coding capacity of SIV yet is incompetent for replication. Such recombinant RRV produces abundant SIV particles in infected cells in culture. Monkeys inoculated with one of these recombinant RRV strains became persistently infected, made readily detectable antibodies against the SIV envelope protein, and developed cellular immune responses to all nine SIV gene products.

## Introduction

There are good reasons for believing that development of an effective preventive vaccine against HIV-1 is going to be a very difficult task [1–3]. HIV is able to replicate continuously without relent despite apparently strong humoral and cellular immune responses to the virus. The HIV envelope glycoprotein is shielded with a large amount of carbohydrate and the trimer spike as it exists of the surface of virions is difficult for antibodies to access and difficult for antibodies to block infectivity. HIV-1 is highly variable from one individual to another and even within a single individual evolves to evade ongoing immune responses. The virus encodes a number of gene products that function at least in part to evade intrinsic, innate and adaptive immune responses. And during the course of an infection, HIV-1 gradually destroys CD4^+^ T lymphocytes, a key orchestrator of adaptive immune responses. The inability of infection by one HIV-1 strain to routinely provide protection against superinfection by a different HIV-1 strain supports this perception of great difficulty in development of a protective vaccine [4]. Investigation of a variety of creative, non-standard approaches to a vaccine seem justified given this expected difficulty.

Two particular vaccine approaches have shown the greatest protective effects in monkey studies to date using virulent strains of simian immunodeficiency virus (SIV) for challenge of Indian-origin rhesus monkeys. The first one consists of live-attenuated strains of SIV, such as those deleted of the *nef* gene, which have far and away provided the greatest degree of protection against challenge [5–8]. However, even live attenuated SIV has not provided very good protection against challenge with SIV strains not closely matched in sequence to that of the vaccine strain [9–11]. This last point seems consistent with the inability of infection by one HIV-1 strain to routinely provide protection against superinfection as described in the previous paragraph. The second approach consists of live recombinant forms of a fibroblast-adapted strain of the beta-herpesvirus rhesus cytomegalovirus (CMV). Approximately 50% of macaques vaccinated with these CMV-based vectors manifested complete control of viral replication shortly after SIVmac239 infection [12–14]. The remaining monkeys not protected by this CMV-based vaccine exhibited persisting SIV levels in plasma indistinguishable from those in control, unvaccinated monkeys. Independent recombinant CMV vectors expressing Gag, or Pol, or Env, or a Rev-Tat-Nef fusion protein (RTN) were combined, but Env-specific antibodies were not elicited.

There are a number of potential advantages to use of a recombinant herpesvirus as a vaccine vector. Herpesviruses possess large genomes and can accommodate a large amount of inserted genetic information. Importantly, herpesviruses persist for the lifetime of the infected host and immune responses to their encoded proteins persist in an up, on, active fashion for life. Being a DNA virus, any inserted genetic information can be expected to remain relatively stable for prolonged periods. Furthermore, there are eight distinct human herpesviruses from which to choose, each with distinct target cells for replication, sites of persistence, and composition of genes. A live-attenuated strain of the human alpha herpesvirus varicella zoster virus is part of childhood immunization programs in many countries [15].

Here we describe the construction and properties of a recombinant gamma-herpesvirus (the rhesus monkey rhadinovirus, RRV) containing a near-full-length genome of SIV capable of expressing 96.7% of its protein products.

## Results

### Expression of SIV-nfl in rRRV-infected rhesus fibroblasts

**Fig 1:**
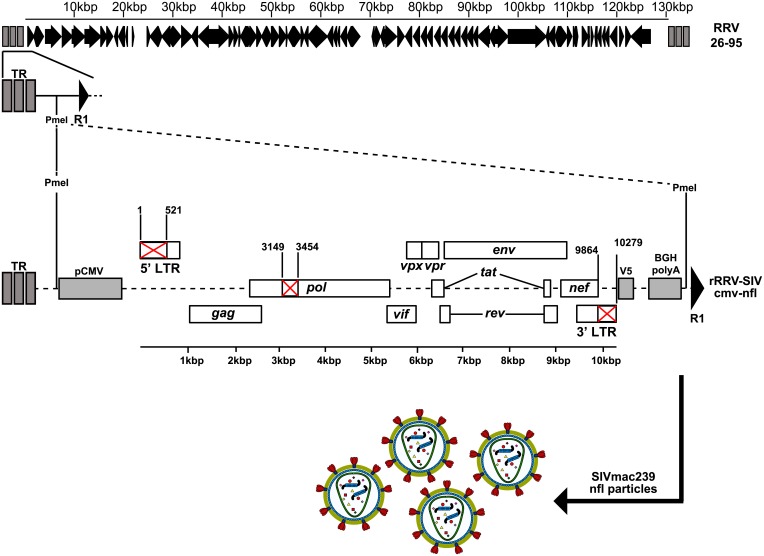
Position of the SIVcmv-nfl expression cassette in recombinant RRV. A near full-length genome (nfl) sequence of SIVmac239 with a 520 bp deletion (red X) in the 5’ Long Terminal Repeat (LTR) region, a deletion in the *pol* region spanning 306 bp, corresponding to the active site of the reverse transcriptase, and a 414 bp deletion in the 3’ LTR region, was generated via Gibson cloning. Numbering system corresponds to that of Regier and Desrosiers [16]. Subsequently, utilizing overlaps containing PmeI restriction sites, the cytomegalovirus immediate-early enhancer and promoter (pCMV), the SIV-nfl sequence containing a C-terminal V5-tagged *nef* open reading frame (ORF), and the bovine growth hormone (BGH) polyA signal were inserted between the left terminal repeats (TR) and the first ORF R1 of RRV via Gibson cloning. Full length recombinant RRV was obtained from subsequent transfection of overlapping cosmid clones [21].

The complete SIVmac239 proviral genome including both LTRs is 10,279 base pairs [16]. For the construction of near-full-length (nfl) recombinants using a different promoter/enhancer region, we eliminated nucleotides 1–521 from the left LTR in the numbering system of Regier and Desrosiers (Fig 1) [16]. We also eliminated nucleotides 9,864–10,279 from the right LTR (Fig 1). The remaining sequences retain the RNA start site in the left LTR and the overlap region with *nef* in the right LTR (Fig 1). A V5 tag was added to the end of *nef*, followed by the BGH poly A addition site (Fig 1). In order to definitively obviate replication competence, a 306-nucleotide in-frame deletion was introduced into *pol* to remove the active site of the reverse transcriptase enzyme (Fig 1). These changes retain 96.7% of the coding capacity of the SIV genome. In one recombinant (r) construct (rRRV-SIVnfl-cmv), a CMV promoter/enhancer region was placed just upstream of the SIV sequences. In a second construct, the SIV promoter/enhancer region was used by restoring the 1–521 nucleotides of the left LTR (rRRV-SIVnfl-ltr). In a third construct, a promoter construct consisting of the promoter for RRV ORF26 (p26) and the promoter for the RRV Poly Adenylated Nuclear RNA (PAN) was inserted just upstream of the SIV sequences (rRRV-SIVnfl-dual). ORF26 encodes a RRV capsid protein and is made late during lytic RRV replication. Conversely, PAN encodes one of the most abundant non-coding RNA transcripts of RRV and is made at the onset of lytic replication [17–20]. These constructions were inserted between the left terminal repeats and the first open reading frame (R1) of the RRV genome using procedures previously described [21–24].

**Fig 2:**
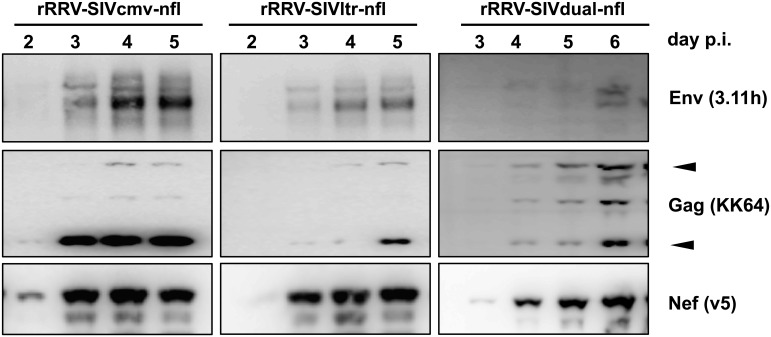
Kinetics of recombinant RRV-mediated SIV-nfl expression. Early passage rhesus fibroblast (RF) cells were grown in 6-well culture plates and the cells in each well were infected with the indicated strains of recombinant rRRV-SIV-nfl. Cells were cultured for the indicated number of days post infection prior to harvest. Subsequently, cells were lysed, and the lysate was subjected to immunoblotting, measuring the expression levels of SIV239 Env, Gag, or Nef. The two bands indicated with arrowheads in the immunoblotting of Gag correspond to p55 and p27. The major Env protein band is gp120; the Nef protein is 34 kDa. For rRRV-SIVltr-nfl the pCMV was replaced with an intact 5’ SIVmac239 LTR. For rRRV-SIVdual-nfl the pCMV was replaced with a promoter construct consisting of the RRV ORF26 promoter (p26) and the RRV Poly Adenylated Nuclear RNA promoter (PAN).

**Fig 3:**
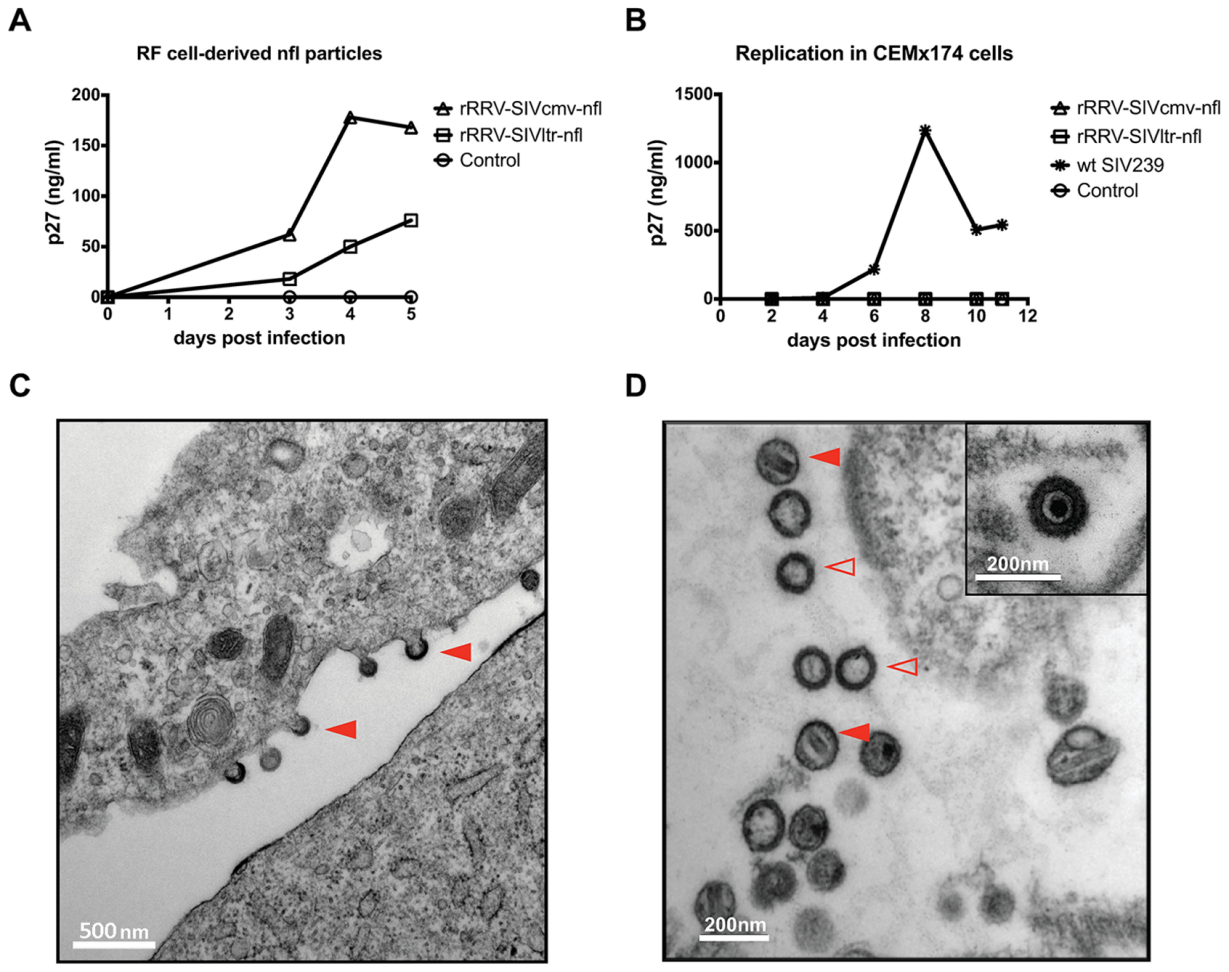
Production of replication-defective SIV particles by rRRV-SIV-nfl. **A)** Early passage rhesus fibroblast (RF) cells were grown in 6-well culture plates and subsequently infected with 3.5x10^9^ genome copies of the indicated recombinant SIV-nfl. The RF cell culture supernatants were collected at the indicated time points post infection and subjected to an antigen capture assay to measure the levels of SIV Gag p27. Supernatant of uninfected RF cells served as a negative control. **B)** Cell culture supernatant was harvested from early passage rhesus fibroblast (RF) cells infected with the respective recombinant RRV-SIV-nfl strains. Subsequently, CEMx174 cells were infected with these supernatants containing 20ng of p27 as measured by an antigen capture assay. An equal amount (20ng of p27) of SIVmac239 wild-type virus produced in HEK239T-cells served as a positive control. The supernatants from the CEMx174 cell cultures were collected at the indicated days post infection and were used in an antigen capture assays measuring p27 levels. Supernatant of uninfected RF cells served as a negative control. **C)** Early passage rhesus fibroblast (RF) cells grown in T75 culture flasks were infected with rRRV-SIVcmv-nfl and harvested at days 4–6 post infection. Subsequently, the cells were fixed with 2.5% glutaraldehyde in sodium cacodylate buffer. Transmission electron microscopic (TEM) images were taken at The Core Electron Microscopy Facility, University of Massachusetts. Shown are SIV particles budding from cell membrane (filled arrowheads). The bar in the lower left corner represents 500 nm **D**) TEM image showing free SIV particles including mature SIV particles (filled arrowheads) containing cylindrical, rod-shaped nucleoid as well as immature particles lacking any nucleoid structures (empty arrowheads). The bar in the lower left corner represents 200 nm. In the inset on the upper right, a TEM image of recombinant RRV derived from a separate image is shown.

To evaluate the expression of SIV proteins, rhesus fibroblasts (RF) permissive for lytic RRV replication were infected with rRRVs expressing SIV-nfl under the control of either the CMV promoter, the SIVmac239 LTR region, or a promoter construct denoted RRV dual promoter, consisting of both p26 and PAN promoters. Cell lysates were prepared and analyzed by immunoblotting for the presence of the particular SIV gene products. All three rRRV vectors produced the early protein Nef and the late structural proteins Gag and Env (Fig 2). The Nef protein was the first detected with all three vectors, suggesting appropriate time-ordered synthesis. Expression levels of the SIV proteins were highest in RF cells infected with the rRRV-SIVcmv-nfl construct. Additionally, the production of the SIV late antigens was first seen at day three post infection in the rRRV-SIVcmv-nfl-infected RF cells. The progression of cytophatic effect was similar with all three viruses, with rRRV-SIVdual-nfl being very slightly slower than the other two, which is why this one goes out to day 6 rather than just day 5 in Fig 2. The observed intracellular expression levels of the SIV proteins were consistent with levels of SIVmac239 Gag p27 detected in RF cell culture supernatants post rRRV infection. RF cells infected with construct rRRV-SIVcmv-nfl released over 150 ng/ml of Gag p27 into the cell-free supernatant (Fig 3A). These impressive levels of p27 are similar to what is observed following replication of SIV in permissive cells [25, 26].

In order to confirm the production and integrity of rRRV-derived SIVnfl virions, transmission electron microscopy (TEM) analysis of rRRV-SIVcmv-nfl-infected RF cells was performed. The TEM images revealed not only RRV particles but also numerous SIV particles of appropriate size and morphology. Free SIV virions released from the cell and virions budding from the surface of the plasma membrane were visualized (Fig 3C & 3D). Of note, some of the extracellular released SIV particles exhibited the cylindrical or rod-shaped nucleoids of mature lentiviral virions. As expected, replication assays performed in CEMx174 cells confirmed that the rRRV-derived SIVnfl particles were replication-incompetent (Fig 3B).

### Infection of rhesus monkeys with rRRV-SIVcmv-nfl

**Fig 4:**
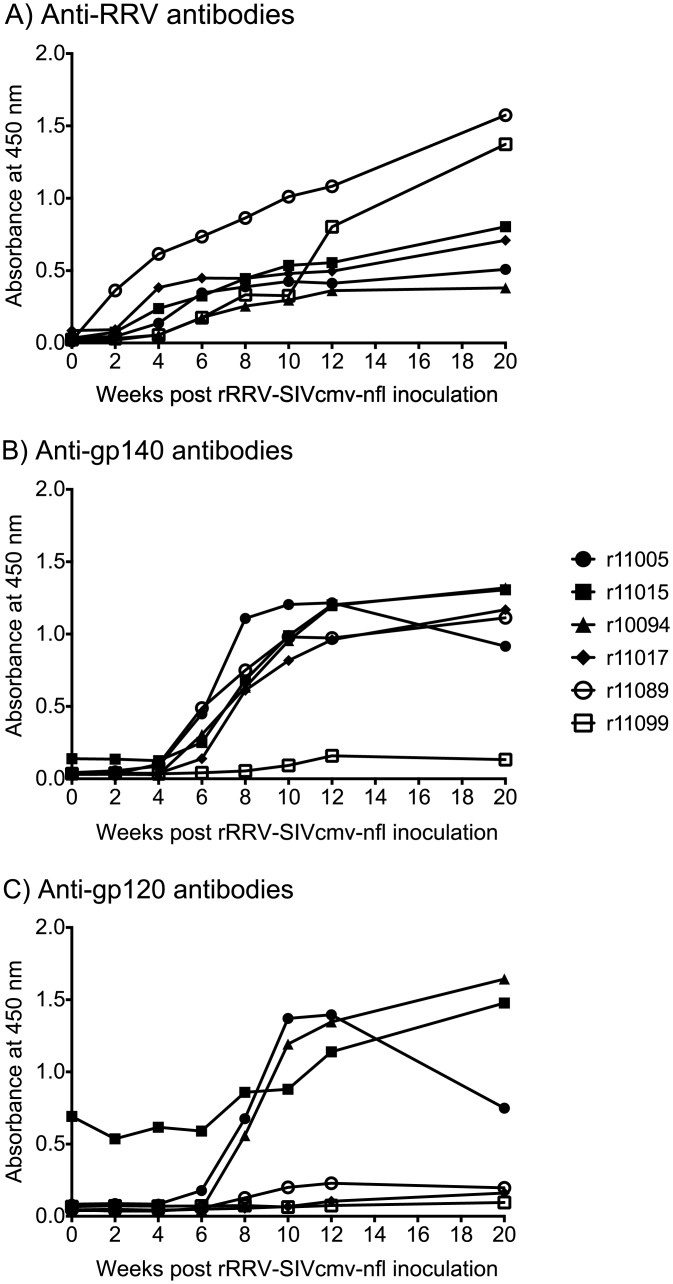
Humoral immune responses following infection of rhesus monkeys with rRRV-SIVcmv-nfl. Sera obtained at the indicated time points following rRRV-SIVcmv-nfl inoculation were diluted 1:20 and tested for the presence of **A)** anti-RRV responses via an ELISA utilizing ELISA plates coated with purified wild type RRV lysate. Reactive antibodies were detected with a horseradish-peroxidase-conjugated anti-rhesus IgG antibody. **B)** Similarly, the sera were tested for the presence of anti-gp140 antibodies via an ELISA using recombinant SIVmac239 gp140 to coat the ELISA plates. Reactive antibodies were detected with a horseradish-peroxidase-conjugated anti-rhesus IgG antibody. **C)** The obtained sera were tested for the presence of anti-gp120 antibodies via an ELISA using recombinant SIVmac239 gp120 to coat the ELISA plates. Reactive antibodies were detected with a horseradish-peroxidase-conjugated anti-rhesus IgG antibody.

**Fig 5:**
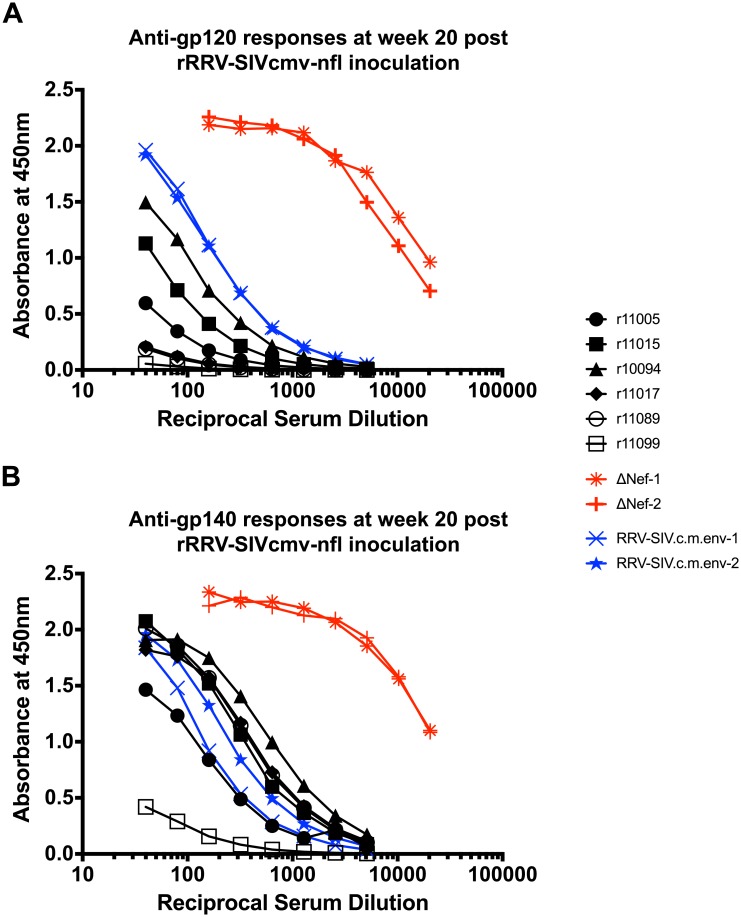
SIV gp120/140 reactivity at week 20 post rRRV-SIVcmv-nfl inoculation. **A)** Sera obtained at week 20 following rRRV-SIVcmv-nfl inoculation were serially diluted and tested for the reactivity to gp120 via an ELISA using recombinant SIVmac239 gp120 to coat the ELISA plates. Subsequently, reactive antibodies were detected with a horseradish-peroxidase-conjugated anti-rhesus IgG antibody. Furthermore, sera of two SIVmac239Δ*nef*-infected animals (week 18 post infection) and sera obtained from two monkeys that had received rRRV expressing a codon-modified version of SIVmac239 gp160 (week 19 post inoculation) were tested [24]. **B)** Similarly, the monkey sera were serially diluted and tested for reactivity to gp140 by ELISA, using recombinant SIVmac239 gp140 to coat the ELISA plates. Reactive antibodies were detected with a horseradish-peroxidase-conjugated anti-rhesus IgG antibody.

Since the rRRV-SIVcmv-nfl construct resulted in high levels of SIV protein expression *in vitro*, we set out to evaluate its ability to infect, persist, and elicit anti-SIV immune responses *in vivo*. Six RRV seronegative rhesus macaques were inoculated intravenously with 10^9^ genome copies of rRRV-SIVcmv-nfl (Table 1). Enzyme-linked immunosorbet assays (ELISAs) were performed to monitor the development of anti-RRV and anti-SIV Env antibodies following the inoculation. Anti-RRV antibodies emerged in all animals by week 6 post rRRV-SIVcmv-nfl inoculation and steadily increased in the ensuing weeks (Fig 4A). Anti-Env antibodies were characterized based on their ability to bind gp140 and gp120. Vaccine-induced gp140-binding antibodies were first detected at week 6 post vaccination and sharply increased afterward (Fig 4B). Except for monkey r11099, all animals maintained high, stable levels of gp140-specific antibodies for the 20 weeks of measurement post rRRV-SIVcmv-nfl inoculation (Fig 4B). Vaccine-induced gp120-binding antibodies were also first detected at week 6 post vaccination, although only two macaques (r11005 and r10094) exhibited a similar surge in antibody levels as was observed for gp140-binding antibodies (Fig 4C). Vaccine-elicited gp120-binding antibodies were still increasing in r10094 and r11015 at week 20 post vaccination, the last follow up (Fig 4C). By comparison, these responses remained at low levels in the remaining rRRV-SIVcmv-nfl vaccinated monkeys. Serial dilutions of week 20 sera were also used to assess vaccine-induced gp140- and gp120-binding antibodies. As references, these analyses included sera from two macaques that had been infected with SIVmac239Δ*nef* for 18 weeks and two monkeys that had been inoculated with a rRRV encoding codon-modified SIVmac239 gp160 (rRRV-SIVc.m.-env) for 19 weeks [24]. The SIVmac239Δ*nef* vaccinees exhibited the highest levels of both gp140- and gp120-binding antibodies (Fig 5A & 5B). While vaccination with rRRV-SIVc.m.-env resulted in greater levels of gp120-binding antibodies than those achieved with rRRV-SIVcmv-nfl (Fig 5A), reactivity to gp140 was comparable among recipients of both rRRV constructs (Fig 5B).

**Table 1:** Characteristics of research animals.

Animal	Sex	Age (yrs)^a^	Relevant MHC-I allele
r11005	Male	4.1	
r11015	Male	4	
r10094	Male	4.2	
r11017	Female	3.9	
r11089	Male	3.2	Mamu-A*01
r11099	Male	3.1	Mamu-A*01

^a^ Age on the day of inoculation

**Fig 6:**
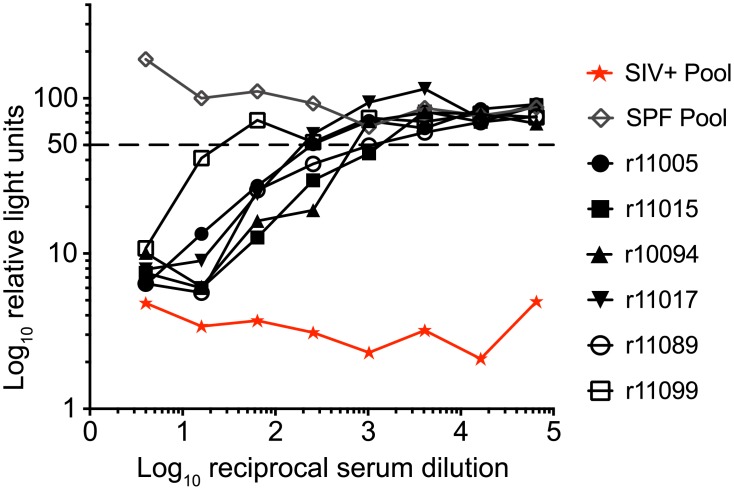
SIVmac316 neutralization titers at week 20 post rRRV-SIVcmv-nfl inoculation in rhesus monkeys. Sera from the rRRV-SIVcmv-nfl-inoculated monkeys taken at week 20 post inoculation were serially diluted and subjected to neutralization assays against SIVmac316 utilizing TZM-bl cells. A pool of sera from SIVmac239-infected monkeys served as a positive control and pooled sera from specific pathogen free (SPF) monkeys as a negative control. The dashed line indicates 50% relative light units representing 50% neutralization activity.

We also evaluated neutralizing activity against SIVmac316 and SIVmac239 using sera obtained at week 20 post rRRV-SIVcmv-nfl administration (Fig 6 and Table 2). SIVmac316 is an easy-to-neutralize derivative of SIVmac239 [27, 28]. Sera from all monkeys were capable of neutralizing SIVmac316 with ID_50_ titers ranging from 1:16 to 1:794 (Fig 6; Table 2). Little or no neutralizing activity was detected against the difficult-to-neutralize SIVmac239 clone.

**Table 2:** Serum neutralization of SIVmac316 at week 20 post rRRV-SIVcmv-nfl vaccination^a^.

ID_50_ titers	
Animal	SIVmac316
r11005	152
r11015	794
r10094	469
r11017	192
r11089	296
r11099	16
SPF^b^	<4
Positive pool^c^	>50,000

^a^ The ID_50_ titers are defined as the serum dilution that results in 50% reduction in SIV infectivity, as measured by the TZM-bl assay.

^b^ SPF, pooled sera of specific pathogen free monkeys.

^c^ Positive pool, pooled sera of SIVmac239-infected monkeys.

**Fig 7:**
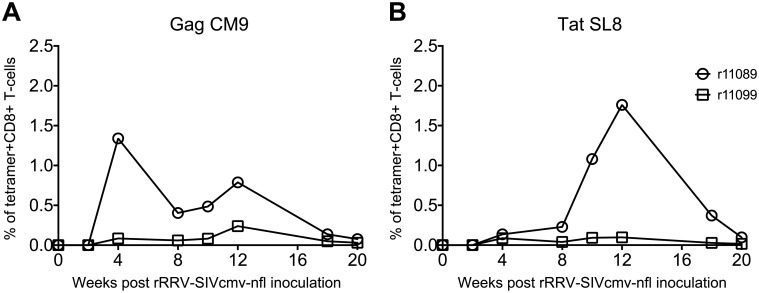
rRRV-SIVcmv-nfl vaccination elicits Gag CM9- and Tat SL8-specific CD8+ T-cell responses in Mamu-A*01+ macaques. Fluorescently-labeled Mamu-A*01 tetramers folded with peptides corresponding to the Gag CM9 (A) or Tat SL8 (B) epitopes were used to monitor the ontogeny of vaccine-induced CD8+ T-cell responses in the two Mamu-A*01+ rRRV-SIVcmv-nfl-inoculated monkeys (r11089 and r11099).

Vaccine-induced SIV-specific cellular immune responses in peripheral blood mononuclear cells (PBMC) were also evaluated. Two of the rRRV-SIVcmv-nfl-vaccinated monkeys (r11089 and r11099) expressed the major histocompatibility complex class I (MHC-I) allele Mamu-A*01, which allowed us to track vaccine-induced SIV-specific CD8+ T-cells by fluorochrome-labeled MHC-I tetramer staining [29]. Mamu-A*01 tetramers folded with peptides corresponding to the immunodominant Gag CM9 (amino acids 181–189) and Tat SL8 (amino acids 28–35) were chosen for this analysis. Vaccine-induced Gag CM9-specific CD8+ T-cells were first detected in both animals at week 4 post vaccination, when they reached a peak of 1.3% of peripheral CD8+ T-cells in monkey r11089 (Fig 7A). In animal r11099, by comparison, these Gag-specific CD8+ T-cell responses reached their highest frequency (0.3%) at week 12 post vaccination (Fig 7A). Low levels of vaccine-elicited Tat SL8-specific CD8+ T-cells were detected in both animals at week 4 post vaccination (Fig 7B). While these responses reached a peak of 1.8% of peripheral CD8+ T-cells in r11089 at week 12 post rRRV-SIVcmv-nfl inoculation, they remained at a low frequency in r11099 until week 20 (Fig 7B). By that time, CD8+ T-cell responses against both Gag CM9 and Tat SL8 had decayed considerably in the two Mamu-A*01+ vaccinees (Fig 7A & 7B).

**Fig 8:**
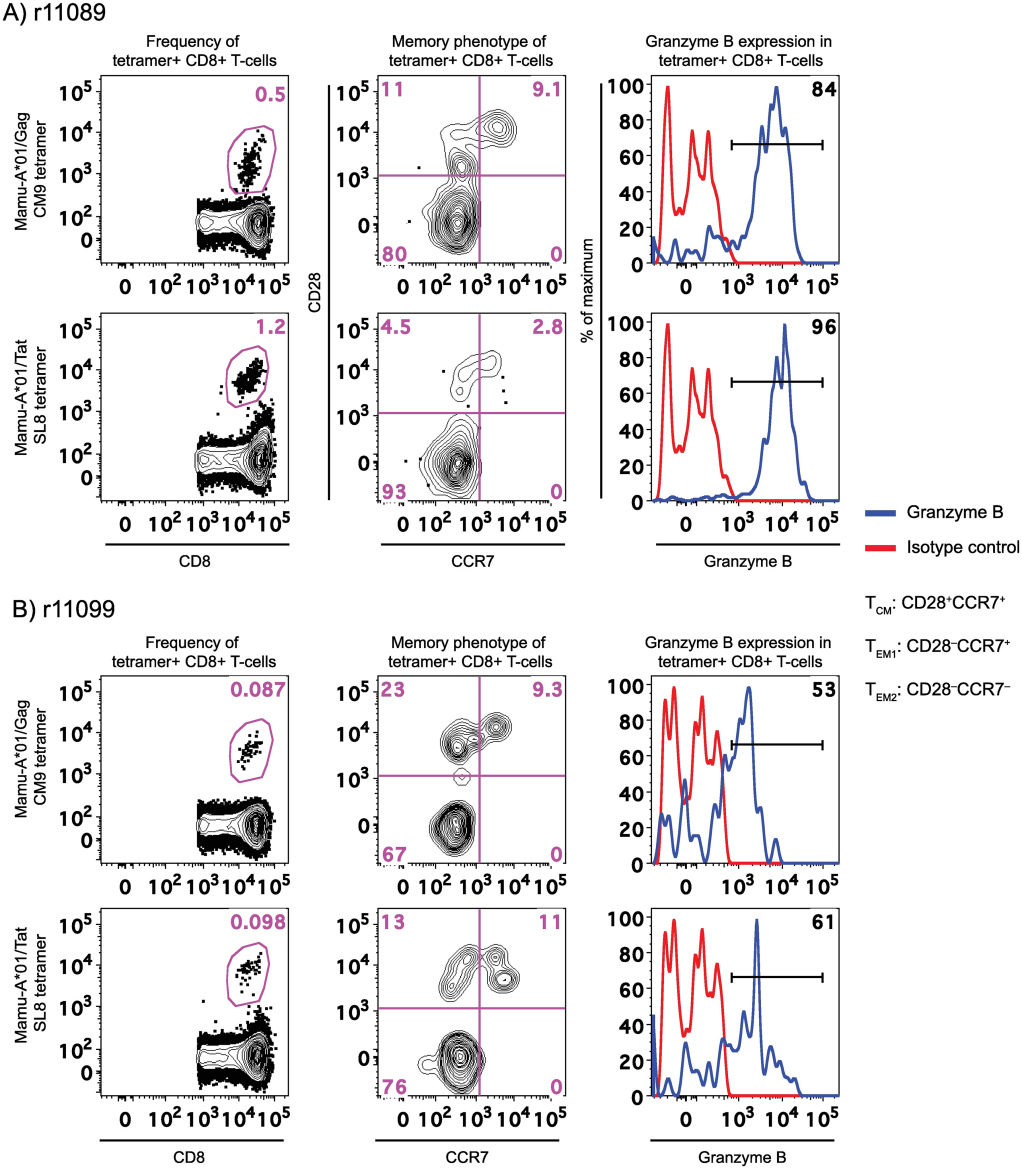
Memory phenotype of vaccine-induced CD8+ T-cell responses in Mamu-A*01+ rRRV-SIVcmv-nfl vaccinees. The frequencies of Mamu-A*01/Gag CM9 and Mamu-A*01/Tat SL8 tetramer+ CD8+ T-cells in PBMC at week 10 post rRRV-SIVcmv-nfl inoculation are shown in the left panels for r11089 (A) and r11099 (B). The middle panels show the delineation of memory subsets within the tetramer+ gate based on the differential expression of CD28 and CCR7. Three subsets were identified: central memory (T_CM_; CD28+CCR7+), transitional memory (T_EM1_; CD28+CCR7-), and effector memory (T_EM2_; CD28-CCR7-). The histograms in the right panels show the levels of granzyme B expressed by tetramer+ CD8+ T-cells (blue lines). The red lines correspond to tetramer+ CD8+ T-cells stained with an isotype-matched control monoclonal antibody.

**Fig 9:**
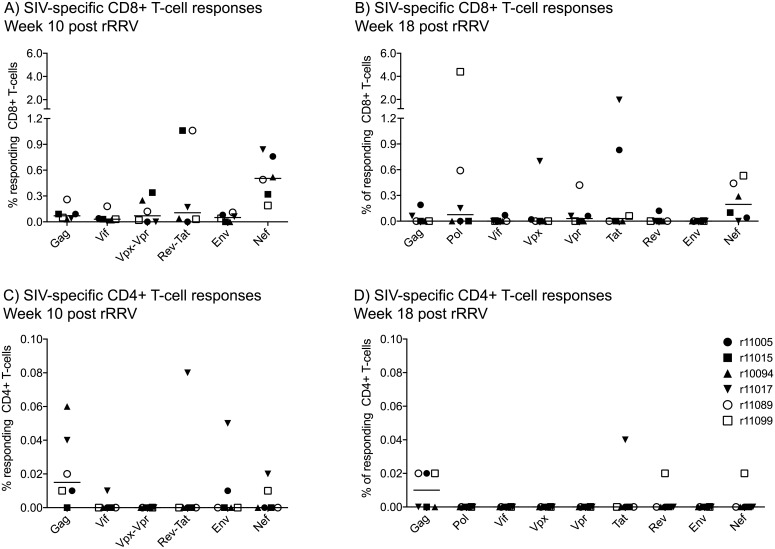
Intracellular cytokine staining analysis of vaccine-induced SIV-specific T-cell responses in rRRV-SIVcmv-nfl-inoculated macaques. The magnitude and specificity of vaccine-elicited CD8+ (A) and CD4+ (B) T-cell responses were measured in PBMC by ICS using pools of peptides (15mers overlapping by 11 amino acids) spanning the appropriate SIVmac239 proteins. In the week 10 assay (A & C), Vpx and Vpr peptides were grouped in a single pool and so were the Rev and Tat peptides. Pol peptides were omitted in the week 10 assay. In the week 18 assay (B & D), 1–3 peptide pools corresponding to individual SIVmac239 proteins were used as stimuli. The percentages of responding CD8+ T-cells or CD4+ T-cells displayed were calculated by adding the frequencies of positive responses producing any combination of three immunological functions (IFN-γ, TNF-α, and CD107a). Lines represent medians and each symbol corresponds to one vaccinee.

We also combined MHC-I tetramer staining with multi-color flow cytometry to evaluate the memory phenotype of vaccine-elicited SIV-specific CD8+ T-cells at week 10 post SIVcmv-nfl inoculation. This analysis relied on the differential expression of CD28 and CCR7 by central memory (T_CM_; CD28+CCR7+), transitional memory (T_EM1_; CD28+CCR7-), and terminally differentiated effector memory (T_EM2_; CD28-CCR7-) CD8+ T-cells in rhesus macaques [30]. The vast majority of Gag CM9- and Tat SL8-specific CD8+ T-cells in r11089 exhibited either the T_EM2_ or T_EM1_ signature, consistent with the persistent nature of the rRRV-SIVcmv-nfl vector (Fig 8A). The low frequencies of tetramer+ CD8+ T-cells in r11099 at week 10 post vaccination made it difficult to accurately delineate the memory phenotype of vaccine-elicited CD8+ T-cells in this animal (Fig 8B). However, based on the few tetramer+ CD8+ T-cells analyzed, T_EM2_ and T_EM1_ subsets appeared to predominate (Fig 8B). Most tetramer+ CD8+ T-cells in r11089 also expressed high levels of the cytotoxicity-associated molecule Granzyme B (Fig 9A). A smaller fraction of tetramer+ CD8+ T-cells in r11099 also appeared to express Granzyme B, albeit at low levels (Fig 8B).

To further evaluate the breadth of vaccine-elicited SIV-specific T-cell responses in the rRRV-SIVcmv-nfl-inoculated monkeys, we carried out intracellular cytokine staining (ICS) assays in PBMC at weeks 10 and 18 post rRRV-SIVcmv-nfl inoculation. Peptide pools corresponding to eight of the nine SIV proteins were used in the first assay–Pol was the only protein absent from that analysis. Vaccine-elicited CD8+ T-cell responses against all eight SIV proteins were detected, especially against Nef (Fig 9A). SIV-specific CD4+ T-cells were also detected at this time point, although they focused on Gag and were present at much lower frequencies (Fig 9B). To further characterize the breadth of vaccine-elicited T-cell responses, we repeated the ICS assay at week 18 using separate pools of peptides corresponding to each of the nine SIV gene products. Nef remained the most frequently targeted viral protein by vaccine-elicited CD8+ T-cells, although abundant Pol- and Tat-specific CD8+ T-cells were also present at this time point (Fig 9C). CD8+ T-cell reactivity against the Vif and Env pools was either negative or at borderline levels in most animals, even though responses against these two proteins were measured in at least one animal at week 10 post rRRV-SIVcmv-nfl inoculation (Fig 9C). The frequency of vaccine-elicited SIV-specific CD4+ T-cells at week 18 was even lower than that measured at week 10 (Fig 9B & 9D). In sum, despite the high animal-to-animal variability in these ICS assays, these data illustrate the capacity of rRRV-SIVcmv-nfl to elicit T-cell responses against all nine SIV gene products.

## Discussion

Herpesviruses have a number of potential advantages when being considered as vectors for vaccine delivery. Herpesviruses have large DNA genomes and can potentially accommodate significant amounts of inserted genetic information. That potential advantage has been borne out in our study described here in that 9,343 base pairs of genetic information have been successfully inserted. Being a DNA virus, inserts can be expected to be reasonably stable in the absence of direct repeats in the insert and with the absence of severe selective disadvantage. Furthermore, herpesviruses persist for the life of the infected individual and immune responses to their proteins persist in an up, on, active fashion for life. This is important when considering vaccine approaches for HIV/AIDS since immunological memory will probably never be enough to contain HIV-1; once a memory response kicks in for a previously-vaccinated, subsequently-infected individual, HIV-1 will employ its vast array of immune evasion and other strategies to allow continuous viral replication. Our results indeed indicate that rRRV-SIVnfl established persistent infection in RRV seronegative monkeys. Antibody responses increased to high levels and persisted through the 20 weeks of analysis. Similarly, cellular responses persisted to one extent or another in the 20 weeks of follow-up analyses. Also, the responding CD8+ T cells exhibited an effector memory phenotype consistent with recurrent antigen production. In contrast to the unconventionally MHC-restricted CD8+ T_EM_ responses elicited by the 68.1 rhesus CMV vaccine developed by Hansen *et al*. [12–14], rRRV-SIVnfl-vaccinated macaques developed CD8+ T-cells capable of recognizing immunodominant SIV epitopes restricted by classical MHC class I molecules.

We are not aware of any live vector system that has attempted what we have described here, i.e. insertion of a nearly complete SIV or HIV genome capable of expression of all nine viral gene products. Ourmanov *et al*. have described insertion of *gag-pol* and *env* genes of SIV into a single recombinant modified vaccinia Ankara (rMVA) vector and the ability of the proteins expressed from this construct to assemble into SIV virion particles [31]. However, six of the SIV genes are not present in this rMVA vector and the infection of monkeys is not persistent.

While live attenuated strains of HIV-1 are not likely to be tried in people anytime soon, finding other ways to mimic them would seem to be a worthwhile goal. The characteristics of the rRRV-SIVnfl strains described here parallel what many consider to be important features for the relative success of live attenuated SIV in monkeys. The infection appears to be persistent, probably lifelong; >95% of the SIV proteome is naturally expressed; virion particles are formed in abundance; and CD8+ T cell responses are maintained indefinitely in an effector-differentiated state. Can we reasonably expect rRRV-SIVnfl to perform as well as live attenuated SIV in monkey vaccine/challenge experiments, particularly since the magnitude of anti-SIV immune responses with rRRV-SIVcmv-nfl fell considerably short of what is seen with live-attenuated SIV infection? It is important to remember in this regard that we have so far tested only one strain of rRRV-SIVnfl in monkeys and that the level of transgene expression in cultured cells is not always predictive of the magnitude of the immune response to that transgene product. For example, a rRRV vector with a CMV promoter/enhancer region driving expression of an SIV *env* gene with an expression-optimized codon usage expressed high levels of Env protein in cultured cells but failed to elicit detectable anti-Env antibodies *in vivo* [22]. In contrast, rRRV expressing a version of SIV *env* with a sub-optimal codon usage capable of being induced by the RRV transinducer ORF57 elicited readily-detectable anti-Env antibodies in infected monkeys [24]. Consequently, no matter how rRRV-SIVcmv-nfl performs in monkey vaccine/challenge experiments, a variety of rRRV-SIVnfl vector designs will need to be compared for the magnitude, persistence and nature of the immune responses to the SIV products and their ability to protect against SIV challenge. Furthermore, there may be ways of enhancing the protective effects of rRRV-SIVnfl, for example by priming or boosting regimens.

## Materials and methods

### Research animals and ethics statement

The details regarding animal welfare described herein are either similar or identical to those published recently [32]. “The Indian rhesus macaques (*Macaca mulatta*) utilized in this study were housed at the Wisconsin National Primate Research Center (WNPRC). All animals were cared for in accordance with the guidelines of the Weatherall report and the principles described in the National Research Council’s Guide for the Care and Use of Laboratory Animals under a protocol approved by the University of Wisconsin Graduate School Animal Care and Use Committee” (animal welfare assurance no. A3368-01; protocol no. G005022) [33]. “Furthermore, the macaques in this study were managed according to the animal husbandry program of the WNPRC, which aims at providing consistent and excellent care to nonhuman primates at the center. This program is employed by the Colony Management Unit and is based on the laws, regulations, and guidelines promulgated by the United States Department of Agriculture (e.g., the Animal Welfare Act and its regulations, and the Animal Care Policy Manual), Institute for Laboratory Animal Research (e.g., Guide for the Care and Use of Laboratory Animals, 8^th^ edition), Public Health Service, National Research Council, Centers for Disease Control, and the Association for Assessment and Accreditation of Laboratory Animal Care International. The nutritional plan utilized by the WNPRC is based on recommendations published by the National Research Council. Specifically, macaques were fed twice daily with 2050 Teklad Global 20% Protein Primate Diet and food intake was closely monitored by Animal Research Technicians. This diet was also supplemented with a variety of fruits, vegetables, and other edible objects as part of the environmental enrichment program established by the Behavioral Management Unit. Paired/grouped animals exhibiting stereotypical and/or incompatible behaviors were reported to the Behavioral Management staff and managed accordingly. All primary enclosures (i.e., stationary cages, mobile racks, and pens) and animal rooms were cleaned daily with water and sanitized at least once every two weeks.” Lights were on a 12:12 diurnal schedule. Vaccinations were performed under anesthesia (Ketamine administered at 5–12 mg/kg depending on the animal) and all efforts were made to minimize suffering. Euthanasia was performed at the end of the study or whenever an animal experienced conditions deemed distressful by one of the veterinarians at the WNPRC. All euthanasia were performed in accordance with the recommendations of the Panel on Euthanasia of the American Veterinary Medical Association and consisted of an IV overdose (greater than or equal to 50 mg/kg or to effect) of sodium pentobarbital or equivalent, as approved by a clinical veterinarian, preceded by ketamine (at least 15 mg/kg body weight) given by the intramuscular (IM) route. Additional animal information, including MHC-I, age at the beginning of study, and sex, is shown in Table 1.

### Cell culture

Early passage rhesus fibroblasts (RFs) were cultured and maintained in Dulbecco’s modified Eagle medium (DMEM, Thermo Fisher Scientific), supplemented with 20% fetal calf serum (Thermo Fisher Scientific), and primocin (InvivoGen). TZM-bl cells (ATCC) were cultivated in DMEM DMEM medium (Thermo Fisher Scientific), supplemented with 10% fetal calf serum (Thermo Fisher Scientific), and primocin (InvivoGen). CEMx174 cells (NIH AIDS Reagent Program) were cultivated in RPMI 1640 medium (Thermo Fisher Scientific), supplemented with 10% fetal calf serum (Thermo Fisher Scientific), and primocin (InvivoGen).

### Generation of recombinant rhesus monkey rhadinovirus

A plasmid containing proviral SIVmac239 DNA served as a template for the generation of a near full-length genome (nfl) sequence of SIVmac239. Two polymerase chain reactions (PCR) led to two SIVmac239 sequence fragments. The primers used were forward primer; ACTTAAGCTTGGTACCGAGCTCGGATCCTCGCTCTGCGGAGAGGCTGGC and reverse primer; GAGTTCCTTTGACTGTAAAACTCCTGCAGGGTGTGGTATTCC, as well as forward primer; GGAATACCACACCCTGCAGGAGTTTTACAGTCAAAGGAACTC and reverse primer; CCACTGTGCTGGATATCTGCAGAATTCGCGAGTTTCCTTCTTGTCAGC. Subsequently, using the PCR-derived BamHI and EcoRI overlaps the SIV-nfl sequence was Gibson cloned (New England BioLabs) into expression plasmid pcDNA6/V5 His A (Thermo Scientific), hereby generating a SIVnfl sequence containing a 520 bp deletion in the 5’ Long Terminal Repeat (LTR) region, a deletion in the *pol* region spanning 306 bp, corresponding to the active site of the reverse transcriptase, and a 414 bp deletion in the 3’ LTR region. Furthermore, the stop-codon after the *nef* open reading frame (ORF) was deleted resulting in a pcDNA6-derived V5-tag following *nef*. A subsequent PCR using the pcDNA6-SIV-nfl plasmid as a template, the forward primer; AGGTACTAGTCCGGCGCCCCGTTTAAACTGACACCTACTCAGACAATGCGAT and reverse primer; ACTATGTGTTACTACTAGTTGTTTAAACTGCTTCGCGATGTACGGGCCAGAT yielded a construct comprised of the cytomegalovirus immediate-early enhancer and promoter (pCMV), the SIV-nfl sequence containing a C-terminal V5-tagged *nef* ORF, and the bovine growth hormone (BGH) polyA signal. Subsequently, utilizing PmeI restriction site overlaps, the SIVnfl construct was cloned into cosmid ah28dA/H between the left terminal repeats (TR) and the first ORF R1 of RRV via Gibson assembly (New England BioLabs). Full length Recombinant RRV was made via co-transfection of five overlapping cosmids as previously described [21]. Recombinant RRV-infected RF culture supernatants were harvested, spun down twice at 2000 rcf for 5 min to remove any cell debris, and resulting virus titers were measured via quantitative real-time PCR using a RRV latency-associated nuclear antigen (LANA) specific primer set. The reaction was completed using the TaqMan Fast Virus 1-Step Master Mix (Thermo Fischer Scientific) in a Real-time PCR thermocycler (Thermo Fisher Scientific); Forward primer; ACCGCCTGTTGCGTGTTA, reverse primer; CAATCGCCAACGCCTCAA, reporter; FAM- CAGGCCCCATCCCC. FAM- CAGGCCCCATCCCC. High-titered recombinant RRV stocks were aliquoted and stored frozen.

### Expression of SIV-nfl in RFs

For immunoblotting experiments, 2.0×10^5^ RFs were seeded into wells of a 6-well plate. The next day, cells were infected with 50 μL of a stock of recombinant RRV containing 10^9^ genome copies/ml expressing either SIVcmv-nfl, SIVlrt-nfl, or SIVdual-nfl. Cells were kept in culture up to six days. Every day cells of one well infected with the respective rRRV were harvested up until the cells were exhibiting advanced cytopathic effect.

### Immunoblotting

Cells were harvested, resuspended, and lysed with an NP40-based lysis buffer including a protease inhibitor (Roche). Cell lysates were spun down to remove any cell debris. Subsequently, supernatants were transferred into new tubes and their protein levels were measured and normalized using a bicinchoninic acid protein assay kit (Pierce). Each lysate was mixed with an equal volume of 2x SDS Laemmli sample buffer (Sigma-Aldrich) containing 2-mercaptoethanol. Then, samples were incubated at 97 °C for 10 min and separated by sodium dodecyl sulfate-polyacrylamide gel electrophoresis (SDS-PAGE). The proteins from the gels were transferred to a polyvinylidene difluoride (PVDF) membrane (Bio-Rad) and subjected to immunoblotting. Furthermore, the PVDF membranes were blocked with 1 x PBS containing 5% skim milk briefly and incubated with the appropriate primary antibodies directed against specific SIV antigens overnight at 4 °C. Three wash steps in PBS containing tween were followed by incubation with the appropriate HRP-conjugated secondary antibodies. After a final washing procedure, specific signals were detected with a LAS4000 mini system (GE Healthcare Systems) using SuperSignal West Pico chemiluminescent substrate (Pierce).

### rRRV-SIV-nfl-derived p27 measurements

Two hundred and fifty thousand RF cells were seeded into 6-well culture plates and subsequently infected with a total of 3.5×10^9^ genome copies of SIVcmv-nfl or SIVdual-nfl. Cell culture supernatants were collected daily and all samples were subjected to a commercial antigen capture assay (ABL) to measure the levels of SIVmac239 Gag p27. Supernatant of uninfected RF cells served as a negative control.

### SIV replication assay

Cell culture supernatant from rRRV-SIV-nfl-infected, early passage RFs were taken. Subsequently, CEMx174 cells were infected with these supernatants containing a total of 20 ng of SIVmac239 Gag p27. Gag p27 levels were measured by a commercial antigen capture assay (ABL). Additionally, 20ng of Gag p27 of HEK293T cell-produced wild type SIVmac239 served as a positive control. The supernatants from the CEMx174 cell cultures were collected daily post infection and were subsequently used in an antigen capture assays measuring Gag p27 levels (ABL). Supernatant taken from cultures of uninfected RF cell served as a negative control.

### Transmission electron microscopy

One million early passage RF cells were seeded into T75 culture flasks. The following day, cells were infected with 200 μL containing 2.0×10^8^ genome copies of rRRV-SIVcmv-nfl. cells were then harvested at days four to six post infection. Subsequently, the cells were fixed with 2.5% glutaraldehyde in sodium cacodylate buffer. Subsequent transmission electron microscopic (TEM) images were taken at the Core Electron Microscopy Facility, University of Massachusetts, according to published procedures [34].

### Vaccination with rRRV-SIVcmv-nfl

One milliliter of PBS containing 10^9^ genome copies of rRRV-SIVcmv-nfl was administered to six rhesus macaques intravenously.

### Enzyme-linked immunosorbent assays (ELISA)

Sera from all research animals were tested for the presence of gp120-binding antibodies. ELISA plates were coated with recombinant gp120 of SIVmac239 (Immune Technology) for 1 h at 37 °C and the plates were then washed with PBS Tween20 (Sigma-Aldrich). Subsequently, the ELISA plates were blocked with PBS containing 5% of skim milk (Bio-Rad). The monkey sera were diluted 1:20 in blocking buffer and applied to the ELISA plate. Then, ELISA plates were incubated for 1 h at 37 °C and the plates washed again. Subsequently, an HRP-conjugated goat anti-rhesus IgG H+L antibody (SouthernBiotech) was added and the ELISA plates incubated for one h at 37 °C. Sequentially, TMB substrate (SouthernBiotech) and stop solution (SouthernBiotech) were added. Lastly, the absorbance at 450 nm per well was read in a microplate reader (PerkinElmer).

Similarly, sera from all six research animals were tested for the presence of antibodies against SIVmac239 gp140 by coating the ELISA plates with homologous purified protein and probing the samples with a horseradish-peroxidase-conjugated anti-rhesus IgG antibody (SouthernBiotech).

All sera were also screened for the presence of anti-RRV antibodies by coating ELISA plates with purified RRV lysate and subsequently probing of samples (diluted 1:20) with a horseradish-peroxidase-conjugated anti-rhesus IgG antibody (SouthernBiotech).

### SIVmac316 neutralization assays

Sera from all six research animals were screened for neutralization of SIVmac316 utilizing the TZM-bl assay, as described previously [35]. The ID_50_ titer is defined as the reciprocal of the highest dilution of serum that reduced SIVmac316 infectivity by 50%. Values were calculated using the Sigmoidal, 4PL, X is log(concentration) equation in Prism7 (GraphPad Software).

### MHC-I tetramer staining and memory phenotyping of CD8^+^ T-cells

The tetramer staining assays performed as part of the time course analysis of vaccine-induced SIV-specific CD8+ T-cells in Mamu-A*01+ macaques (Fig 7) was done by labeling PBMC with titrated amounts of fluorochrome-conjugated Mamu-A*01/Gag CM9 (MBL International Inc.) or Mamu-A*01/Tat SL8 (Tetramer Core Facility) tetramers [29]. Up to 800,000 PBMC were incubated with the respective tetramers at 37 °C for 1 hr and then stained with fluorochrome-labeled monoclonal antibodies (mAbs) directed against the surface molecules CD3 (clone SP34-2) and CD8α (clone RPA-T8). After a 25-min incubation at RT, the cells were washed and then fixed with PBS containing 2% of paraformaldehyde. Data were acquired with a SORP BD LSR II (BD BioSciences, San Jose, CA) flow cytometer and analyzed with FlowJo software version 9.9.3 (Tree Star, Inc. Ashland, OR). CD3+ T cells were gated within the lymphocyte gate defined by the forward and side-scatter properties. Tetramer positive cell frequencies were determined within the CD3+CD8+ cell population.

The following description of how the memory phenotype and granzyme B content of tetramer+ CD8+ T-cells were determined is either identical or similar to that used in one of our recent publications [36]. “Up to 8.0×10^5^ cells were incubated in the presence of the appropriate fluorochrome-labeled tetramer at 37 °C for 1 h and then stained with monoclonal antibodies (mAbs) directed against the surface molecules CD3 (clone SP34-2), CD8α (clone RPA-T8), CD28 (clone 28.2), CCR7 (clone 150503), CD14 (clone M5E2), CD16 (clone 3G8), and CD20 (clone 2H7). Amine-reactive dye (ARD; Live/DEAD Fixable Aqua Dead Cell Stain; Life Technologies) was also added to this mAb cocktail. After a 25-min incubation at room temperature, we treated the cells with BD FACS Lysing Solution (BD Biosciences) for 10 min and subsequently washed them with “Wash Buffer” (Dulbecco’s PBS with 0.1% BSA and 0.45 g/L NaN_3_). Cells were permeabilized by treatment with “Perm buffer” [1X BD FACS Lysing Solution 2 (Beckton Dickinson) and 0.05% of Tween-20 (Sigma-Aldrich)] for 10 min. Cells were then washed once and stained with a Granzyme B-specific mAb (clone GB12). After a 30-min incubation in the dark at room temperature, cells were washed and stored at 4 °C until acquisition. Samples were acquired using FACS DIVA version 6 on a Special Order Research Product BD LSR II apparatus equipped with a 50-mW 405-nm violet, a 100-mW 488-nm blue, and a 30-mW 635-nm red laser.

We used FlowJo 9.6 (Treestar, Inc.) to analyze data. First, we gated on diagonally clustered singlets by plotting forward scatter height (FSC-H) versus FSC area (FSC-A) and then side scatter height (SSC-H) versus SSC area (SSC-A). Next, we created a time gate that included only those events that were recorded within the 5^th^ and 90^th^ percentiles and then gated on “dump channel” negative, CD3^+^ cells. At this stage, we delineated the lymphocyte population based on its FSC-A and SSC-A properties and subsequently gated on CD8^+^ cells. After outlining tetramer^+^ cells, we conducted our memory phenotyping analysis within this gate. Cells stained with fluorochrome-labeled mAbs of the same isotypes as the anti-Granzyme B, anti-CD28, and anti-CCR7 mAbs guided the identification of the memory subsets within the tetramer^+^ population. Based on this gating strategy, the tetramer frequencies shown in Fig 9 correspond to percentages of live CD3^+^ CD8^+^ tetramer^+^ lymphocytes.”

### Intracellular cytokine staining (ICS) assay

Pools of peptides (15mers overlapping by 11 amino acids) spanning all nine SIVmac239 gene products were used for T-cell stimulation in the week 18 assay. Given the large size of Gag, Pol, and Env, the peptides covering these polyproteins were divided in 2, 3, and 2 pools, respectively. Peptides spanning each of the remaining accessory (Vpr, Vpx, Vif, and Nef) and regulatory (Rev and Tat) proteins were grouped in individual pools. For the week 10 assay, Vpx and Vpr peptides were grouped in a single pool, as were the Rev and Tat peptides. Pol peptides were not used in the week 10 assay. The final assay concentration of each 15mer was 1.0 μM. The following description on how the ICS assays were set up is nearly identical to that used in our recent publications [32]. “PBMC obtained from the research animals were stimulated with the appropriate pools of SIVmac239 peptides in RPMI 1640 medium supplemented with GlutaMax (Life Technologies), 10% FBS (VWR), and 1% antibiotic/antimycotic (VWR) containing co-stimulatory mAbs against CD28 and CD49d for 9 h at 37 °C in an incubator with a 5.0% CO_2_ concentration. Moreover, a phycoerythrin-conjugated mAb specific for CD107a was included in the assay. Brefeldin A (Biolegend, Inc.) and GolgiStop (BD Biosciences) were added to all tubes 1 h into the incubation time to inhibit any protein transport. Surface molecules of cells were stained as mentioned above and cells were fixed with a 2% paraformaldehyde solution. In addition to the same mAbs against CD14, CD16, and CD20 and the ARD reagent described above, the surface staining master mix also included mAbs against CD4 (clone OKT4; Biolegend, Inc.) and CD8 (clone RPA-T8; Biolegend, Inc.). Cells were permeabilized by resuspending them in “Perm Buffer” (1× BD FACS lysing solution 2 (Beckton Dickinson) and 0.05% Tween-20 [Sigma-Aldrich]) for 10 min and subsequently washed with Wash Buffer. Cells were then incubated with mAbs against CD3 (clone SP34-2), IFN-γ (clone 4S.B3), TNF-α (clone Mab11), and CD69 (clone FN50) for 1 h in the dark at RT. After this incubation was completed, the cells were washed and subsequently stored at 4 °C until acquisition. The data were analyzed by gating first on live CD14–CD16–CD20–CD3+ lymphocytes and then on cells expressing either CD4 or CD8 but not both markers. Functional analyses were conducted within these two compartments. Cells were considered positive for IFN-γ, TNF-α, or CD107a only if they co-expressed these molecules with CD69, a marker of recent activation. Once the appropriate gates were created, we employed the Boolean gate platform to generate a full array of possible combinations, equating to 8 response patterns when testing three functions (2^3^ = 8). Leukocyte activation cocktail (LAC; BD Pharmingen)-stimulated cells stained with fluorochrome-labeled mAbs of the same isotypes as those against IFN-γ, TNF-α, and CD107a guided the identification of positive populations. We used two criteria to determine if responses were positive. First, the frequency of events in each Boolean gate had to be at least two-fold higher than their corresponding values in background-subtracted negative-control tests. Second, the Boolean gates for each response had to contain ≥10 events. The magnitude of responding CD4+ or CD8+ T-cells was calculated by adding the frequencies of positive responses producing any combination of IFN-γ, TNF-α, and CD107a. All calculations, including background subtraction and evaluation of the frequencies of responding cells, were performed with Microsoft Excel.”

## References

[1] Desrosiers RC. Strategies used by human immunodeficiency virus that allow persistent viral replication. Nat Med. 1999;5:723–725. doi: 10.1038/10439 10395309 10.1038/10439

[2] Desrosiers RC. Prospects for an AIDS vaccine. Nat Med. 2004;10:221–223. doi: 10.1038/nm0304-221 14991035 10.1038/nm0304-221

[3] Evans DT, Desrosiers RC. Immune evasion strategies of the primate lentiviruses. Immunol Rev. 2001;183:141–158. 11782254 10.1034/j.1600-065x.2001.1830112.x

[4] Piantadosi A, Chohan B, Chohan V, McClelland RS, Overbaugh J. Chronic HIV-1 infection frequently fails to protect against superinfection. PLoS Pathog. 2007;3:e177 doi: 10.1371/journal.ppat.0030177 18020705 10.1371/journal.ppat.0030177 PMC2077901

[5] Almond N, Kent K, Cranage M, Rud E, Clarke B, Stott EJ. Protection by attenuated simian immunodeficiency virus in macaques against challenge with virus-infected cells. Lancet. 1995;345:1342–1344. 7752758 10.1016/s0140-6736(95)92540-6

[6] Daniel MD, Kirchhoff F, Czajak SC, Sehgal PK, Desrosiers RC. Protective effects of a live attenuated SIV vaccine with a deletion in the nef gene. Science. 1992;258:1938–1941. 1470917 10.1126/science.1470917

[7] Johnson RP, Lifson JD, Czajak SC et al Highly attenuated vaccine strains of simian immunodeficiency virus protect against vaginal challenge: inverse relationship of degree of protection with level of attenuation. J Virol. 1999;73:4952–4961. 10233957 10.1128/jvi.73.6.4952-4961.1999 PMC112539

[8] Wyand MS, Manson KH, Garcia-Moll M, Montefiori D, Desrosiers RC. Vaccine protection by a triple deletion mutant of simian immunodeficiency virus. J Virol. 1996;70:3724–3733. 8648707 10.1128/jvi.70.6.3724-3733.1996 PMC190248

[9] Manrique J, Piatak M, Lauer W et al Influence of mismatch of Env sequences on vaccine protection by live attenuated simian immunodeficiency virus. J Virol. 2013;87:7246–7254. doi: 10.1128/JVI.00798-13 23637396 10.1128/JVI.00798-13 PMC3700272

[10] Reynolds MR, Weiler AM, Piaskowski SM et al Macaques vaccinated with simian immunodeficiency virus SIVmac239Delta nef delay acquisition and control replication after repeated low-dose heterologous SIV challenge. J Virol. 2010;84:9190–9199. doi: 10.1128/JVI.00041-10 20592091 10.1128/JVI.00041-10 PMC2937616

[11] Wyand MS, Manson K, Montefiori DC, Lifson JD, Johnson RP, Desrosiers RC. Protection by live, attenuated simian immunodeficiency virus against heterologous challenge. J Virol. 1999;73:8356–8363. 10482586 10.1128/jvi.73.10.8356-8363.1999 PMC112853

[12] Hansen SG, Vieville C, Whizin N et al Effector memory T cell responses are associated with protection of rhesus monkeys from mucosal simian immunodeficiency virus challenge. Nat Med. 2009;15:293–299. doi: 10.1038/nm.1935 19219024 10.1038/nm.1935 PMC2720091

[13] Hansen SG, Ford JC, Lewis MS et al Profound early control of highly pathogenic SIV by an effector memory T-cell vaccine. Nature. 2011;473:523–527. doi: 10.1038/nature10003 21562493 10.1038/nature10003 PMC3102768

[14] Hansen SG, Sacha JB, Hughes CM et al Cytomegalovirus vectors violate CD8+ T cell epitope recognition paradigms. Science. 2013;340:1237874. 10.1126/science.1237874 PMC3816976 23704576

[15] Quinlivan M, Breuer J. Clinical and molecular aspects of the live attenuated Oka varicella vaccine. Rev Med Virol. 2014;24:254–273. doi: 10.1002/rmv.1789 24687808 10.1002/rmv.1789

[16] Regier DA, Desrosiers RC. The complete nucleotide sequence of a pathogenic molecular clone of simian immunodeficiency virus. AIDS Res Hum Retroviruses. 1990;6:1221–1231. 2078405 10.1089/aid.1990.6.1221

[17] Alexander L, Denekamp L, Knapp A, Auerbach MR, Damania B, Desrosiers RC. The primary sequence of rhesus monkey rhadinovirus isolate 26–95: sequence similarities to Kaposi’s sarcoma-associated herpesvirus and rhesus monkey rhadinovirus isolate 17577. J Virol. 2000;74:3388–3398. 10708456 10.1128/jvi.74.7.3388-3398.2000 PMC111840

[18] Nealon K, Newcomb WW, Pray TR, Craik CS, Brown JC, Kedes DH. Lytic replication of Kaposi’s sarcoma-associated herpesvirus results in the formation of multiple capsid species: isolation and molecular characterization of A, B, and C capsids from a gammaherpesvirus. J Virol. 2001;75:2866–2878. doi: 10.1128/JVI.75.6.2866-2878.2001 11222712 10.1128/JVI.75.6.2866-2878.2001 PMC115913

[19] Song MJ, Li X, Brown HJ, Sun R. Characterization of interactions between RTA and the promoter of polyadenylated nuclear RNA in Kaposi’s sarcoma-associated herpesvirus/human herpesvirus 8. J Virol. 2002;76:5000–5013. doi: 10.1128/JVI.76.10.5000-5013.2002 11967316 10.1128/JVI.76.10.5000-5013.2002 PMC136175

[20] Tycowski KT, Shu MD, Borah S, Shi M, Steitz JA. Conservation of a triple-helix-forming RNA stability element in noncoding and genomic RNAs of diverse viruses. Cell Rep. 2012;2:26–32. doi: 10.1016/j.celrep.2012.05.020 22840393 10.1016/j.celrep.2012.05.020 PMC3430378

[21] Bilello JP, Morgan JS, Damania B, Lang SM, Desrosiers RC. A genetic system for rhesus monkey rhadinovirus: use of recombinant virus to quantitate antibody-mediated neutralization. J Virol. 2006;80:1549–1562. doi: 10.1128/JVI.80.3.1549-1562.2006 16415030 10.1128/JVI.80.3.1549-1562.2006 PMC1346924

[22] Bilello JP, Manrique JM, Shin YC et al Vaccine protection against simian immunodeficiency virus in monkeys using recombinant gamma-2 herpesvirus. J Virol. 2011;85:12708–12720. doi: 10.1128/JVI.00865-11 21900170 10.1128/JVI.00865-11 PMC3209374

[23] Bischof GF, Magnani DM, Ricciardi M et al Use of a Recombinant Gamma-2 Herpesvirus Vaccine Vector against Dengue Virus in Rhesus Monkeys. J Virol. 2017;91 10.1128/JVI.00525-17 PMC5533908 28592531

[24] Shin YC, Bischof GF, Lauer WA, Desrosiers RC. Importance of codon usage for the temporal regulation of viral gene expression. Proc Natl Acad Sci U S A. 2015;112:14030–14035. doi: 10.1073/pnas.1515387112 26504241 10.1073/pnas.1515387112 PMC4653223

[25] Morrison HG, Kirchhoff F, Desrosiers RC. Evidence for the cooperation of gp120 amino acids 322 and 448 in SIVmac entry. Virology. 1993;195:167–174. doi: 10.1006/viro.1993.1357 8317093 10.1006/viro.1993.1357

[26] Stansell E, Canis K, Haslam SM, Dell A, Desrosiers RC. Simian immunodeficiency virus from the sooty mangabey and rhesus macaque is modified with O-linked carbohydrate. J Virol. 2011;85:582–595. doi: 10.1128/JVI.01871-10 20962077 10.1128/JVI.01871-10 PMC3014205

[27] Means RE, Greenough T, Desrosiers RC. Neutralization sensitivity of cell culture-passaged simian immunodeficiency virus. J Virol. 1997;71:7895–7902. 9311879 10.1128/jvi.71.10.7895-7902.1997 PMC192146

[28] Mori K, Ringler DJ, Kodama T, Desrosiers RC. Complex determinants of macrophage tropism in env of simian immunodeficiency virus. J Virol. 1992;66:2067–2075. 1548752 10.1128/jvi.66.4.2067-2075.1992 PMC288997

[29] Gonzalez-Nieto L, Domingues A, Ricciardi M et al Analysis of Simian Immunodeficiency Virus-specific CD8+ T-cells in Rhesus Macaques by Peptide-MHC-I Tetramer Staining. J Vis Exp. 2016 10.3791/54881 PMC5226457 28060314

[30] Picker LJ, Reed-Inderbitzin EF, Hagen SI et al IL-15 induces CD4 effector memory T cell production and tissue emigration in nonhuman primates. J Clin Invest. 2006;116:1514–1524. doi: 10.1172/JCI27564 16691294 10.1172/JCI27564 PMC1459071

[31] Ourmanov I, Brown CR, Moss B et al Comparative efficacy of recombinant modified vaccinia virus Ankara expressing simian immunodeficiency virus (SIV) Gag-Pol and/or Env in macaques challenged with pathogenic SIV. J Virol. 2000;74:2740–2751. 10684290 10.1128/jvi.74.6.2740-2751.2000 PMC111764

[32] Martins MA, Shin YC, Gonzalez-Nieto L et al Vaccine-induced immune responses against both Gag and Env improve control of simian immunodeficiency virus replication in rectally challenged rhesus macaques. PLoS Pathog. 2017;13:e1006529 doi: 10.1371/journal.ppat.1006529 28732035 10.1371/journal.ppat.1006529 PMC5540612

[33] Weatherall D. The use of non-human primates in research. FRS FMedSci; 2006:152 p.

[34] McKenzie CW, Craige B, Kroeger TV et al CFAP54 is required for proper ciliary motility and assembly of the central pair apparatus in mice. Mol Biol Cell. 2015;26:3140–3149. doi: 10.1091/mbc.E15-02-0121 26224312 10.1091/mbc.E15-02-0121 PMC4569307

[35] Montefiori DC. Measuring HIV neutralization in a luciferase reporter gene assay. Methods Mol Biol. 2009;485:395–405. doi: 10.1007/978-1-59745-170-3_26 19020839 10.1007/978-1-59745-170-3_26

[36] Martins MA, Tully DC, Cruz MA et al Vaccine-Induced Simian Immunodeficiency Virus-Specific CD8+ T-Cell Responses Focused on a Single Nef Epitope Select for Escape Variants Shortly after Infection. J Virol. 2015;89:10802–10820. doi: 10.1128/JVI.01440-15 26292326 10.1128/JVI.01440-15 PMC4621113

